# Nitrogen-Doped Porous Co_3_O_4_/Graphene Nanocomposite for Advanced Lithium-Ion Batteries

**DOI:** 10.3390/nano9091253

**Published:** 2019-09-03

**Authors:** Huihui Zeng, Baolin Xing, Lunjian Chen, Guiyun Yi, Guangxu Huang, Ruifu Yuan, Chuanxiang Zhang, Yijun Cao, Zhengfei Chen

**Affiliations:** 1 Henan Key Laboratory of Coal Green Conversion, College of Chemistry and Chemical Engineering, Henan Polytechnic University, Jiaozuo 454003, China; 2 Henan Province Industrial Technology Research Institute of Resources and Materials, Zhengzhou University, Zhengzhou 450001, China; 3 Laboratory of Polymer Materials and Engineering, Ningbo Institute of Technology, Zhejiang University, Ningbo 315100, China

**Keywords:** Co_3_O_4_ nanoparticles, anthracite-derived graphene, nanocomposite, lithium-ion batteries

## Abstract

A novel approach is developed to synthesize a nitrogen-doped porous Co_3_O_4_/anthracite-derived graphene (Co_3_O_4_/AG) nanocomposite through a combined self-assembly and heat treatment process using resource-rich anthracite as a carbonaceous precursor. The nanocomposite contains uniformly distributed Co_3_O_4_ nanoparticles with a size smaller than 8 nm on the surface of porous graphene, and exhibits a specific surface area (120 m^2^·g^−1^), well-developed mesopores distributed at 3~10 nm, and a high level of nitrogen doping (5.4 at. %). These unique microstructure features of the nanocomposite can offer extra active sites and efficient pathways during the electrochemical reaction, which are conducive to improvement of the electrochemical performance for the anode material. The Co_3_O_4_/AG electrode possesses a high reversible capacity of 845 mAh·g^−1^ and an excellent rate capacity of 587 mAh·g^−1^. Furthermore, a good cyclic stability of 510 mAh·g^−1^ after 100 cycles at 500 mA·g^−1^ is maintained. Therefore, this work could provide an economical and effective route for the large-scale application of a Co_3_O_4_/AG nanocomposite as an excellent anode material in lithium-ion batteries.

## 1. Introduction

Rechargeable batteries play a crucial part in portable electronic devices and electromobiles as energy storage and conversion devices [1]. Lithium-ion batteries (LIBs) have drawn extensive concern owing to their high energy density, long cycling life, high power, and safety [2,3]. In LIBs, the anode material is one of many factors that can affect their ultimate performance [4,5]. However, the current graphite anode with a low theoretical capacity (372 mAh·g^−1^) limits the performance improvement of LIBs [6]. Therefore, it is highly necessary to explore new advanced anode material for a new generation of LIBs.

Many transition metal oxides [7,8,9,10] show potential as promising anode candidates for LIBs by reason of their better theoretical capacity, high power density, and easy accessibility [11,12]. Among these materials, Co_3_O_4_ is a potential anti-ferromagnetic p-type semiconductor with a spinel crystal structure, which can coordinate with eight lithium ions per single lattice and deliver a higher theoretical capacity of 890 mAh·g^−1^ [13,14]. Unfortunately, pure Co_3_O_4_ as an anode is subjected to a poor cycling stability and rate capability because of its poor electronic conductivity, and the pulverization or agglomeration of primitive particles during charging/discharging processes [15]. Generally, engineering nanostructured Co_3_O_4_ and preparing Co_3_O_4_ composites are considered to heighten the structural stability and enhance the properties of the Co_3_O_4_ anode. It is universally accepted that the nanostructure and morphology of the Co_3_O_4_ are significant to avoid electrical isolation of the electrode due to the Li_2_O produced during the conversion process [16,17]. In line with this hypothesis, a series of studies on engineering various nanostructured Co_3_O_4_, such as nanoparticles [18], nanosheets [19], and microfibers [20], have been subsequently carried out. Although the property of these nanostructured Co_3_O_4_ as an anode in LIBs has been improved, the complex process and high cost for preparing these Co_3_O_4_ are hurdles for practical applications.

Co_3_O_4_/carbon composites, such as Co_3_O_4_/carbon nano-onions [21], Co_3_O_4_/carbon nanotube [22], Co_3_O_4_/porous carbon [23], and Co_3_O_4_/graphene hybrid [4], are potentially anode materials for LIBs. Among numerous materials, Co_3_O_4_/graphene composites have attracted extensive research due to their unique structural advantages [15]: firstly, the superior electronic conductivity of graphene can shorten the path length to facilitate faster lithium ion and electron diffusion; secondly, graphene has a large theoretical capacity (744 mAh·g^−1^) as it can provide additional reactive sites for lithium ion storage [24]; thirdly, the structural flexibility and large specific surface area of graphene can offer adequate void spaces to relieve the large volume expansion of Co_3_O_4_ nanoparticles during charging/discharging processes; and lastly, the agglomeration of Co_3_O_4_ nanoparticles may be prevented when they are evenly dispersed on the surface of graphene. However, employing 2D structural graphene as a substrate does not significantly improve the electrochemical property of Co_3_O_4_ because of the easy aggregation of the graphene nanosheets on account of the van der Waals interactions among these nanosheets [25]. As a result, some research effort has been made to construct 3D Co_3_O_4_/graphene using copper foam as a template in order to overcome this shortcoming [26]. However, this process is very complicated, limiting its use in commercial applications.

In line with this, coal may be a desirable material to prepare porous graphene, because it contains abundant polyaromatic structures like sp^2^ bonding structures, which are cross-linked via some oxygen-containing functional groups [27]. There are some literature reports on the preparation of graphene electrodes with a high performance from various coals, such as 3D graphene from coal tar pitch [28] and graphene/Mn_3_O_4_ composites from coal-derived graphite [29]. Recently, our group investigated the preparation of porous graphene by a graphitization process, followed by an oxidation-thermal treatment approach [30]. These works have demonstrated that using coal as a raw material to prepare porous graphene is feasible, but the practical application of graphene is still limited due to its high irreversible capacity [2].

In this work, we report an economical and effective approach to fabricate a nitrogen-doped porous Co_3_O_4_/anthracite-derived graphene (Co_3_O_4_/AG) nanocomposite using the earth-abundant and low-cost anthracite as a carbonaceous precursor. The anthracite was firstly graphitized to obtain a highly lamellar structure and was then converted into anthracite-derived graphene oxide (AGO) by a modified Hummers method. Next, the AGO was used as a carbon source to synthesize the nitrogen-doped porous Co_3_O_4_/AG nanocomposites by self-assembly, followed by a heat treatment method. The crystal structure and composition, porous morphology structure, surface chemical property, and electrochemical performances of these samples were systematically investigated. This nanocomposite contained crystalline Co_3_O_4_ nanoparticles, which were evenly dispersed on the surface of porous graphene and had a large surface area, displaying a much better electrochemical performance as an anode in LIBs.

## 2. Materials and Methods

### 2.1. Preparation of AGO

The powdered anthracite sample from the Ningxia region in China was firstly carbonized at 1000 °C under argon atmosphere, followed by further graphitization at 2800 °C, to obtain synthetic graphite. Then, the flocculent AGO was prepared via a modified Hummers method following our previous work [30].

### 2.2. Synthesis of Co_3_O_4_/AG Nanocomposite

**Figure 1:**
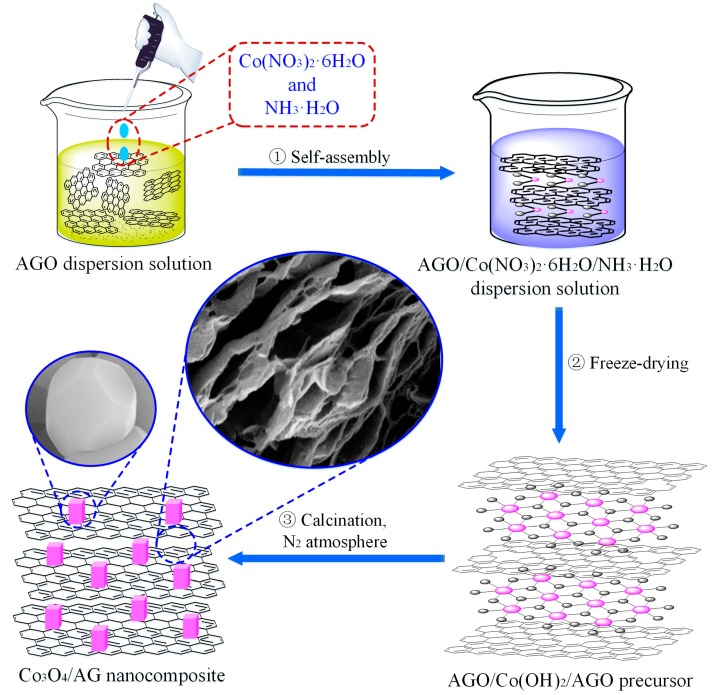
Schematic illustration of the synthetic process of the Co_3_O_4_/AG nanocomposite.

The Co_3_O_4_/AG nanocomposite was synthesized via self-assembly, followed by a heat treatment method. In detail, 1 g of AGO was completely dispersed in 150 mL aqueous solution by ultrasound treatment for 1 h. After that, 50 mL of 10 mg/mL Co(NO_3_)_2_·6H_2_O was slowly dropped into the AGO suspension drop-wise under continuous stirring, followed by the addition of 10 mL 28 wt. % ammonium hydroxide (NH_3_·H_2_O). This mixture was continuously stirred for 2 h to produce cobalt hydroxide and to allow the self-assembly Co_3_O_4_ nanoparticles on AGO. After the reaction, the Co_3_O_4_/AG nanocomposite precursor was dried through freeze-drying and then further calcined at 500 °C for 2 h under N_2_ atmosphere, and the final Co_3_O_4_/AG nanocomposite was obtained after grinding. The synthetic process of the Co_3_O_4_/AG nanocomposite is illustrated in Figure 1. For comparison purposes, Co_3_O_4_ nanoparticles were prepared via the same process, without the addition of AGO, and anthracite-derived graphene (AG) was obtained using AGO as a raw material by the same calcination method.

### 2.3. Material Characterization

The X-ray diffraction (XRD, Bruker, Karlsruhe, Germany) patterns of Co_3_O_4_, AG, and the Co_3_O_4_/AG nanocomposite were recorded by a D8 Advance instrument (Cu Kα radiation, λ = 0.15418 nm) at the range of 10–80°, and the Raman spectra were acquired on an inVia Raman spectroscope (Renishaw, London, UK, Ar ion laser, λ = 514 nm) from 2400 to 200 cm^−1^. A Quanta FEG 250 scanning electron microscopy (SEM, FEI, Hillsboro, Oregon, USA) and one JEM-2100 transmission electron microscope (TEM, JEOL, Tokyo, Japan) were employed to observe the morphological structure. The N_2_ adsorption measurement was conducted on an Autosorb-iQ-MP instrument (Quantachrome, Norcross, GA, USA) at −196 °C. Meanwhile, the Brunauer–Emmett–Teller (BET) model was applied to evaluate the specific surface area of AG and the Co_3_O_4_/AG nanocomposite. The X-ray photoelectron spectroscopy (XPS, Thermo Fisher Scientific, Waltham, MA, USA) analyses were measured by an Escalab 250Xi instrument (Al Kα radiation, 1486.6 eV) to confirm the chemical composition of samples. Lastly, the thermogravimetric (TG, Netzsch, Bavaria, Germany) analysis of the nanocomposite was studied on a STA409 PC thermogravimetric analyzer under air flow (30–700 °C, 10 °C min^−1^).

### 2.4. Electrochemical Measurements

Electrochemical measurements were evaluated by a coin-type cell (CR2016, Jinghong, Zhengzhou, China). The working electrodes were constructed by mixing 80 wt. % as-prepared samples, 10 wt. % acetylene black, and 10 wt. % polyvinyldene fluoride (PVDF, Aladdin, Shanghai, China) in N-methylpyrrolidone (NMP, Aladdin, Shanghai, China) to form slurry and the mixed slurry was then uniformly painted onto copper foil. After drying at 105 °C under vacuum, some disks with a diameter of 14 mm were obtained from the painted copper foil. Besides, pure lithium foil and a polypropylene microporous film (Celgard 2400) were employed as a separator and counter electrode, and 1M LiPF_6_ in ethylene carbonate/dimethyl carbonate (1:1 vol. ratio) was applied as the electrolyte. A BTS-3000n test instrument (Neware, Shenzhen, China) was employed for galvanostatic charge/discharge measurements, with a voltage window from 0.01 to 3.0 V (vs. Li/Li^+^). Cyclic voltammetry (CV) was carried out on a CHI660D workstation (Chenhua, Shanghai, China). Electrochemical impedance spectroscopy (EIS) was conducted on a Parstat 2273 workstation (Princeton applied research, Oak ridge, TN, USA) in a frequency range of 100 to 0.01 Hz.

## 3. Results and Discussion

### 3.1. Material Characterization

**Figure 2:**
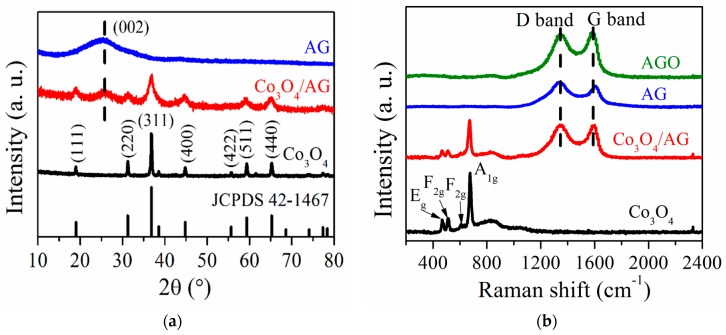
XRD patterns (**a**) and Raman spectra (**b**) of Co_3_O_4_, AG, and the Co_3_O_4_/AG nanocomposite.

The phase compositions of Co_3_O_4_, AG, and the Co_3_O_4_/AG nanocomposite were analyzed by XRD patterns, as shown in Figure 2a. For Co_3_O_4_, the main diffraction peaks at 19.0°, 31.3°, 36.7°, 44.7°, 59.4°, and 65.4° can be respectively assigned to (111), (220), (311), (400), (511), and (440) crystal planes (JCPDS 42-1467) [18]. The Co_3_O_4_/AG nanocomposite contains only the diffraction peaks of Co_3_O_4_ and AG (the (002) peak at around 26°), confirming that the Co_3_O_4_ nanoparticles in the sample have excellent crystallinity and AGO was reduced to AG during the synthetic process of the nanocomposite. Furthermore, the structures of these samples were evaluated by Raman spectra, as demonstrated in Figure 2b. For Co_3_O_4_, the four peaks at 470, 513, 608, and 676 cm^−1^ correspond to the E_g_, F_2g_, F_2g_, and A_1g_ modes of spinel Co_3_O_4_, separately [31]. The AG sample shows a D and G band at around 1343 and 1589 cm^−1^, which correspond to the characteristics of defect sites and a disordered structure, and the features of ordered few-layer graphene, respectively [32]. As expected, the Co_3_O_4_/AG nanocomposite has a combination of peaks from Co_3_O_4_ and AG. Meanwhile, it can be found that the Co_3_O_4_/AG nanocomposite shows a relatively larger intensity ratio of the D band to the G band (I_D_/I_G_ = 1.02) than that of AGO (I_D_/I_G_ = 0.96), indicating that more structural defects exist in the nanocomposite after calcination. However, the I_D_/I_G_ value of the nanocomposite is lower than that of AG (I_D_/I_G_ = 1.18), suggesting that Co_3_O_4_ has impacted the formation of structural defects in AG during the synthetic process of the nanocomposite. It is commonly accepted that some defects in a nanocomposite can provide more active sites, which are conductive to storing more lithium ions for a high capacity [33]. Therefore, the Co_3_O_4_/AG nanocomposite may act as a potential candidate for a high-performance anode in LIBs.

**Figure 3:**
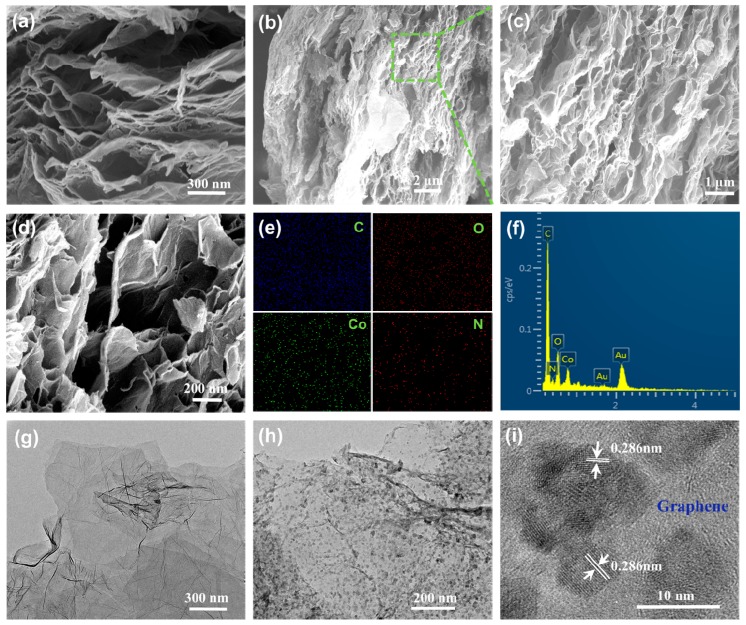
Microstructure characteristics of samples: (**a**) Scanning electron microscopy (SEM) images of AG, (**b**–**d**) SEM images of the Co_3_O_4_/AG nanocomposite, (**e**) and (**f**) elemental mapping and EDS of the Co_3_O_4_/AG nanocomposite, (**g**) and (**h**) transmission electron microscope (TEM) micrographs of AG and the Co_3_O_4_/AG nanocomposite, and (**i**) HRTEM micrograph of the Co_3_O_4_/AG nanocomposite.

The microstructure characteristics of AG and the Co_3_O_4_/AG nanocomposite are exhibited in Figure 3. The AG sample (Figure 3a) shows a continuous wrinkled sheet-like structure, and these nanosheets are interconnected to construct a 3D porous structure with abundant nanoscale pores. After the Co_3_O_4_ nanoparticles were dispersed onto these nanosheets, the morphologies and structure of the nanocomposite could be seen, as shown in Figure 3b–d. The SEM images (Figure 3b,c) at a lower magnification exhibit a lot of wrinkled AG nanosheets with a porous structure and the three-dimensional porous structure in the nanocomposite can be clearly observed at a higher magnification (Figure 3d). The element mapping of the sample (Figure 3e) suggests that the C, O, Co, and N elements are evenly existent in the nanocomposite, suggesting that the finer-grained Co_3_O_4_ are well-dispersed on the surface of AG. The energy dispersive spectrometer (EDS) profile (Figure 3f) confirms that the nanocomposite is only comprised of C, O, Co, and N elements. Besides, the TEM micrograph (Figure 3g) also demonstrates that the AG sample has a wrinkled sheet-like structure composed of several layers. For the TEM image of the nanocomposite in Figure 3h, it can be observed that the Co_3_O_4_ nanoparticles are homogeneously dispersed onto the wrinkled AG nanosheets. The high resolution transmission electron microscope (HRTEM) image (Figure 3i) reveals that the Co_3_O_4_ nanoparticles with a size smaller than 8 nm are distributed on the surface of AG, and the interplanar distance is 0.286 nm, corresponding to the (220) plane of Co_3_O_4_ nanoparticles [34].

**Figure 4:**
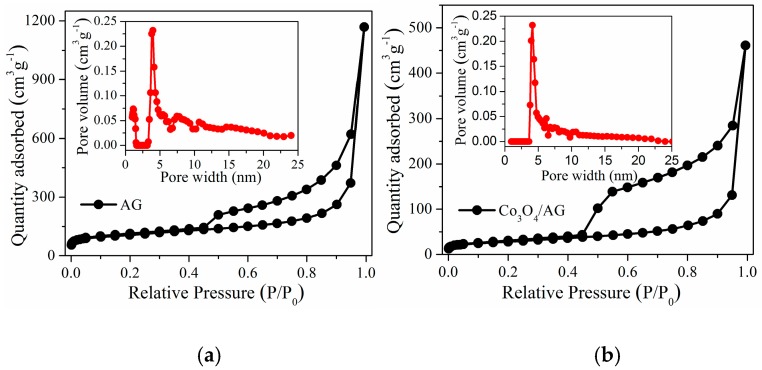
Nitrogen adsorption and desorption isotherms (inset: pore size distribution) of samples: (**a**) AG and (**b**) the Co_3_O_4_/AG nanocomposite.

The nitrogen adsorption and desorption isotherms of AG and the Co_3_O_4_/AG nanocomposite reveal their detailed porous structures, which are shown in Figure 4. These isotherms belong to a typical type IV structure with an obvious hysteresis loop, which indicates that the material contains a large amount of mesopore structures. For the AG sample (Figure 4a), it exhibits a wide mesopore size distribution from 3 to 25 nm and a small number of micropores at around 1.2 nm. After the addition of Co_3_O_4_ nanoparticles, the pore size distribution of the Co_3_O_4_/AG nanocomposite (Figure 4b) narrows down to the region of 3~10 nm, owing to the fact that some of the large pores are filled by Co_3_O_4_ nanoparticles. Accordingly, the specific surface area calculated by the BET model falls from 383 m^2^·g^−1^ for the AG to 120 m^2^·g^−1^ for the Co_3_O_4_/AG nanocomposite. Nevertheless, the specific surface area of this material is still better than that of the pure Co_3_O_4_ [35] and other Co_3_O_4_/graphene composites [36,37,38]. Such a porous structure and large surface of the product can not only protect the electrode from damage due to the volume change of Co_3_O_4_, but also effectively favor the rapid diffusion of lithium ions during the electrochemical reaction, which is conducive to improvement of the performance for the material as an anode in LIBs.

**Figure 5:**
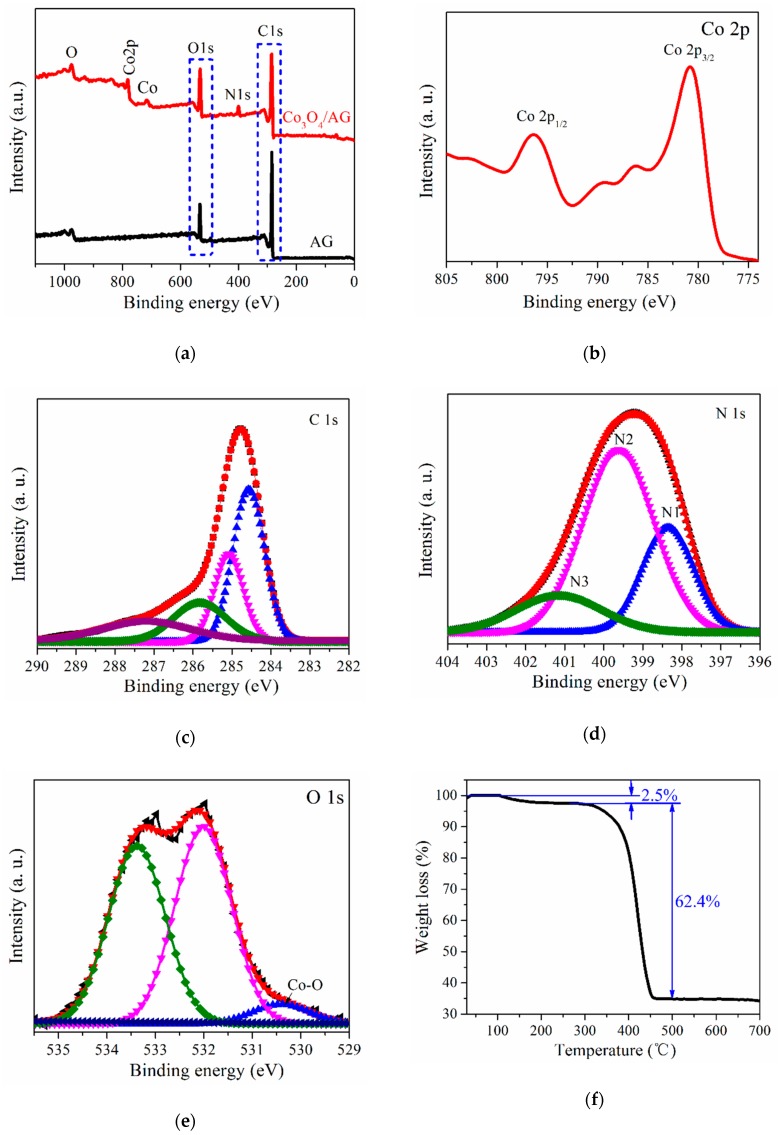
XPS survey spectra and thermogravimetric (TG) curve of samples: (**a**) Wide scan spectra of AG and the Co_3_O_4_/AG nanocomposite; (**b**), (**c**), (**d**), and (**e**) high-resolution Co 2p, C 1s, N 1s, and O 1s spectra of the Co_3_O_4_/AG nanocomposite; (**f**) TG curve of the Co_3_O_4_/AG nanocomposite.

The surface chemistry of AG and the Co_3_O_4_/AG nanocomposite was investigated by XPS measurement, as shown in Figure 5a–e. The survey spectrum of AG (Figure 5a) demonstrates the existence of only C and O elements, whereas that of the nanocomposite contains Co, C, N, and O elements. This suggests that N atoms were introduced to the composite during the synthesis process, which resulted from the excess ammonium hydroxide which reacted with carbon atoms near defects and vacancies or oxygen functional groups in the AGO [39]. From Figure 5d, the N 1s spectrum could be decomposed into three peaks (398.3, 399.6, and 401.2 eV), which could be ascribed to pyridinic (N1), pyrrolic (N2), and graphitic (N3) nitrogen atoms, separately. Meanwhile, XPS analysis shows that 5.4 at. % of nitrogen had been successfully doped into the Co_3_O_4_/AG nanocomposite. Because of its higher electronegativity of N (3.5) compared to C (3.0), the doped N could modify the graphene planar sheet and might play a crucial part in enhancing the electrochemical performance in LIBs [40]. In addition, the two binding energy peaks of Co2p at 796.3 and 780.9 eV shown in Figure 5b are respectively attributed to the spin effect of Co2p_1/2_ and Co2p_3/2_ electrons, which is in line with the reported Co_3_O_4_ [26]. From the high-resolution C1s spectrum (Figure 5c), the deconvoluted four peaks at 284.6, 285.1, 285.8, and 287.2 eV correspond to graphite-like sp^2^ C, C-OH, N-sp^2^ C, and sp^3^ C [41], respectively. The O1s spectrum (Figure 5e) could be deconvoluted into three peaks, and the peaks at 530.4 and 531.9 eV were assigned to the lattice oxygen species in the Co_3_O_4_ and the oxygen in the OH– or C–O–C groups, separately [42]. Besides, the one at 533.4 eV belongs to the Co–O–C bond and C=O groups on the AG surface, indicating that the Co_3_O_4_ nanoparticles in the nanocomposite were anchored on the surface of AG sheets, which has been researched in detail in the literature [43]. Furthermore, the content of AG and Co_3_O_4_ in the Co_3_O_4_/AG nanocomposite was tested by TG measurement in Figure 5f. The weight loss (2.5%) below 150 °C is due to the loss of adsorbed water on the surface of the nanocomposite, and the weight loss at 150–450 °C is due to the decomposition and removal of oxygen functional groups. On the basis of the TG curve, it can be seen that the contents of AG and Co_3_O_4_ in the nanocomposite are about 62.4 wt. % and 35.1 wt. %, respectively [37].

### 3.2. Electrochemical Performances

**Figure 6:**
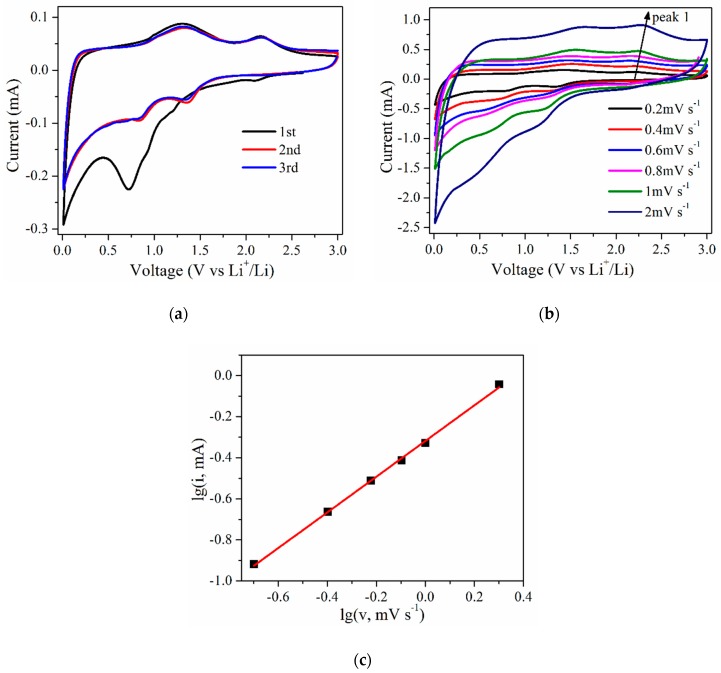
Cyclic voltammetry (CV) behavior of the Co_3_O_4_/AG nanocomposite: (**a**) CV curves at 0.1 mV·s^−1^, (**b**) CV curves at various scan rates from 0.2 to 2 mV·s^−1^, and (**c**) the relationship between the anodic peak current at around 2.20 V and the scan rate.

The CV scans of the Co_3_O_4_/AG nanocomposite at 0.1 mV·s^−1^ over the voltage range from 0.01 to 3.00 V were investigated, as shown in Figure 6a. During the first scan, one broad reduction peak can be found during 0.5–1.0 V, which is the initial reduction of Co_3_O_4_ along with the formation of amorphous Li_2_O and solid electrolyte interphase (SEI) film [23]. The peak close to 0 V is deemed to arise from lithium ion intercalation into AG, indicating that AG in the material was also beneficial for lithium storage. The broad peaks at 1.30 and 2.20 V in the anodic scan were respectively attributed to the delithiation process of AG and the formation of CoO [44]. Electron energy loss spectroscopy (EELS) analysis showed that the white-line intensity ratio (L_3_/L_2_) after the first delithiation process corresponded to the valence state of 2+, which indicates that the oxidation product of metallic cobalt embedded in the Li_2_O matrix is CoO rather than Co_3_O_4_. Meanwhile, such a CoO product was also confirmed by in situ TEM and the electron diffraction (ED) pattern [45]. Therefore, the electrochemical reaction of lithium-ions with Co_3_O_4_ is irreversible during the first cycle. During the second scan, two cathodic peaks at around 0.84 and 1.36 V can be observed, which corresponds to the decomposition process of SEI film and the reduction of CoO [46]. Compared to the peaks of the first scan, the anodic peaks at 1.30 and 2.20 V exhibit little change, suggesting a good reversibility during the charge/discharge reaction. Furthermore, the shape of the third scan is almost identical to that of the second one, which reveals that the cycling stability in the material is built after the first cycle.

To better understand the kinetic process, CV measurements at the scan rate of 0.2 to 2 mV·s^−1^ were carried out. The CV curves of the Co_3_O_4_/AG nanocomposite (Figure 6b) show obvious and well-defined redox peaks, which indicates that the conversion reactions of cobalt oxides with lithium have excellent kinetics [47]. If a redox reaction is controlled by semi-infinite diffusion, the relationship between the peak current (i) and the scan rate (v) is denoted as i = av^b^, where the value of power coefficient b provides insight into the lithium-ion storage mechanism in the electrode [48]. Therefore, it could be obtained from Figure 6c that the logarithm of the current response (i) at 2.20 V for peak 1 exhibits a linear dependence with the logarithm of the sweep rate (v). The electrode kinetics that arose from surface-controlled behavior was confirmed by the value of power coefficient b (0.86), which is beneficial to and may facilitate the lithium-ion insertion/extraction processes [4].

**Figure 7:**
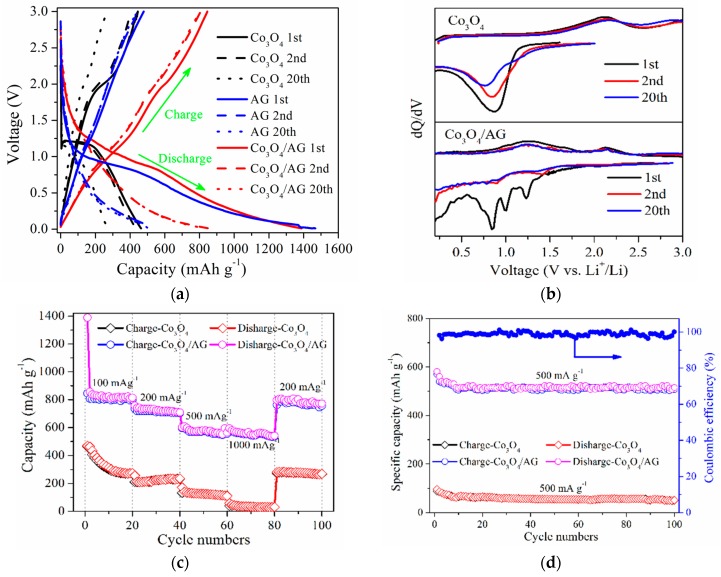
Electrochemical performances of Co_3_O_4_, AG, and the Co_3_O_4_/AG nanocomposite: (**a**) Galvanostatic charge/discharge curves, (**b**) differential capacity curves, (**c**) rate capability at various current densities from 100 to 1000 mA·g^−1^; (**d**) Cycling performance and coulombic efficiency at a current density of 500 mA·g^−1^ for 100 cycles.

Figure 7a shows the galvanostatic charge/discharge behaviors of Co_3_O_4_, AG, and the Co_3_O_4_/AG nanocomposite at a current density of 100 mA·g^−1^ in a voltage range of 0.01 to 3.00 V (vs. Li/Li^+^). The initial discharge and charge capacities of the nanocomposite electrode are 1388 and 845 mAh·g^−1^, respectively, with the initial coulombic efficiency of 60.8%, which are superior to those of the Co_3_O_4_ electrode (463 and 444 mAh·g^−1^, respectively) and importantly, the introduction of Co_3_O_4_ into the composite resulted in lower initial irreversible capacity loss compared to the AG electrode with the initial coulombic efficiency of 32.5%, corresponding to the initial discharge and charge capacities of 1467 and 478 mAh·g^−1^, respectively. As for the Co_3_O_4_ electrode, the polarization (i.e., large voltage hysteresis) between the discharge and charge limited by lithium diffusion kinetics is the main factor of irreversible capacity loss [49]. Moreover, the reversible capacity of the nanocomposite is superior to the reported porous Co_3_O_4_/graphene [50], hollow Co_3_O_4_ nanoparticles [51], and Co_3_O_4_/graphene foams [52]. These suggest that AG with a porous structure can effectively improve the reversible capacity of pure Co_3_O_4_. In addition, the initial capacity loss for the nanocomposite is mainly due to the formative SEI film and the irreversible reaction between lithium-ions and the electrodes [53]. In the subsequent cycles, the Co_3_O_4_/AG nanocomposite likewise displays a much improved charge retention capacity compared to the Co_3_O_4_ and AG electrode.

To understand the structural evolution in the nanocomposite electrode, the corresponding differential capacity curves for the 1st, 2nd, and 20th charge/discharge cycles are exhibited in Figure 7b. For the Co_3_O_4_ electrode, the peak at 1.10 V corresponds to its voltage plateau (about 1.10 V) in the first discharge process in Figure 7a. However, this peak position decreases with increasing cycle numbers, indicating that the capacity of the pure Co_3_O_4_ electrode is unstable. On the other hand, the Co_3_O_4_/AG nanocomposite shows a good reversibility and cyclability during the process after the first cycle. The differential capacity curve of initial discharge shows three peaks: one profound peak at 0.85 V and two smaller peaks at 1.00 and 1.24 V. The former one is mainly due to the formation of SEI film on the nanocomposite surface and the insertion of lithium-ions into the AG, indicating that the AG in the nanocomposite is beneficial to store lithium-ions. The other two peaks correspond to the reduction reaction of lithium-ions with Co_3_O_4_, which indicates that the reduction reaction of Co_3_O_4_ is a multi-step electron capture procedure [54]. Therefore, the reduction reaction can be expressed as the following step [55]:

Co_3_O_4_ + 8Li^+^ + 8e^−^→3Co + 4Li_2_O (1)

During the charge process, there are two anodic peaks, corresponding to the two voltage plateaus at 1.30 and 2.10 V in the first charge curve in Figure 7a, which corresponds to the decomposition process of SEI film and the reduction of CoO. During the subsequent discharge processes, the cathodic peaks shift to around 0.87 and 1.4 V and remain unchanged, which suggests that the reversible redox reaction is gradually built. Additionally, the reversible redox reaction can be expressed as the following steps [56]:

CoO + 2Li^+^ + 2e^−^↔Li_2_O + Co, (2)

The above results can demonstrate that the electrochemical reaction of the Co_3_O_4_/AG nanocomposite electrode is irreversible during the first cycle, but a reversible reaction upon the conversion of Co and CoO embedded in the Li_2_O matrix is built in the subsequent cycles.

The rate capability of these samples at the current densities of 100 to 1000 mA·g^−1^ is displayed in Figure 7c. Compared to Co_3_O_4_, the Co_3_O_4_/AG exhibits a better rate capability, delivering the reversible capacities of 845, 756, 600, and 587 mAh·g^−1^ at current densities of 100, 200, 500, and 1000 mA·g^−1^, respectively. Besides, the performance of this Co_3_O_4_/AG nanocomposite outperformed that of other Co_3_O_4_/graphene composites [57,58]. In particular, the reversible capacity returns to 790 mAh·g^−1^ with the decrease of current density to 200 mA·g^−1^, demonstrating the superior recovery ability of the Co_3_O_4_/AG nanocomposite. Furthermore, the cycling stability of Co_3_O_4_ and Co_3_O_4_/AG at a current density of 500 mA·g^−1^ for 100 cycles is shown in Figure 7d. The Co_3_O_4_/AG exhibits a high initial charge capacity of 570 mAh·g^−1^, which is much higher than that for Co_3_O_4_ (95 mAh·g^−1^). Meanwhile, the capacity of the nanocomposite is almost constant at 510 mAh·g^−1^ in the subsequent cycles, and the coulombic efficiency is maintained at above 96%; however, the capacity of Co_3_O_4_ exhibits a constant decrease from the initial capacity of 95 mAh·g^−1^ to 48 mAh·g^−1^ during the 100 cycles. Compared to the capacity of Co_3_O_4_/carbon aerogel hybrids (478 mAh·g^−1^ at 50 mA·g^−1^), Co_3_O_4_-NP (400 mAh·g^−1^ at 200 mA·g^−1^), the Co_3_O_4_/CC@Gr composite (391 mAh·g^−1^ at 100 mA·g^−1^), and block-Co_3_O_4_/graphene (400 mAh·g^−1^ at 500 mA·g^−1^) [59,60,61,62], the cycling performance of the Co_3_O_4_/AG nanocomposite is comparable and close to the capacity of Co_3_O_4_ nanowire/graphene (500 mAh·g^−1^ at 1 C) [63] and the Co_3_O_4_/graphene composite (600 mAh·g^−1^ at 500 mA·g^−1^) [56].

**Figure 8:**
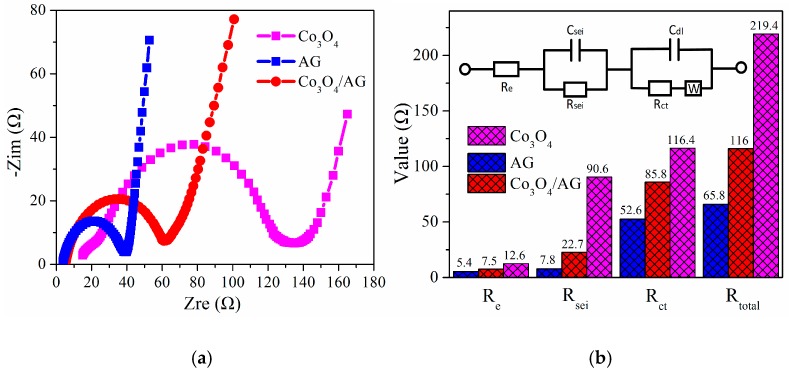
Electrochemical impedance spectroscopy (EIS) behavior of samples: (**a**) Nyquist plots and (**b**) the values of R_e_, R_sei_, R_ct_, and R_total_ simulated by the equivalent circuit for Co_3_O_4_, AG, and the Co_3_O_4_/AG nanocomposite (inset: the whole equivalent circuit for anode materials).

The EIS measurements were applied to obtain insight into the transport kinetics process of the Co_3_O_4_, AG, and Co_3_O_4_/AG electrodes. The Nyquist plots of these samples in Figure 8a have a typical semicircle and an inclined line, corresponding to the SEI film, and charge-transfer and lithium-ion diffusion resistance, respectively [64]. As for the AG electrode, it shows the smallest semicircle and the most oblique line compared to Co_3_O_4_ and Co_3_O_4_/AG electrodes, indicating that the AG electrode has an excellent electronic conductivity. The size of the semicircle for the Co_3_O_4_/AG electrode was smaller than that of the Co_3_O_4_ electrode, which indicates that AG as a substrate can enhance the electronic conductivity of electrode material. In addition, an equivalent electrical circuit was applied to fit the impedance values, and the detailed fitting values are shown in Figure 8b. In brief, the parameters for R_e_, R_sei_, R_ct_, and W represent the resistance of the electrolyte and electrode, the resistance of the SEI film, the charge transfer resistance, and the Warburg impedance related to lithium ion diffusion, separately [33]. Clearly, the R_e_, R_sei_, and R_ct_ values of the Co_3_O_4_/AG electrode are smaller than that of Co_3_O_4_, and it could be found that the R_total_ (116.0 Ω) of the nanocomposite is much lower than that of Co_3_O_4_ (219.4 Ω), which suggests that the introduction of AG as a substrate could improve the enhanced kinetics of lithium-ions and electronic transport in the nanocomposite electrode. These results could demonstrate that the Co_3_O_4_/AG nanocomposite as an anode in LIBs possesses high electrical conductivity and excellent reaction kinetics for lithium ions.

The outstanding electrochemical performance of the Co_3_O_4_/AG nanocomposite could be assigned to two factors: First, the porous nanostructure resulting from AG can shorten the diffusion distance of lithium ions and provide extra active sites to store lithium-ions; meanwhile, a large amount of pores in the AG substrate can relieve the volume change of the Co_3_O_4_ nanoparticles during the charge/discharge reaction. Second, nitrogen doped in the nanocomposite is conducive to improvement of the electrical conductivity for the nanocomposite. Therefore, the Co_3_O_4_/AG nanocomposite is a promising anode material in LIB applications.

## 4. Conclusions

A Co_3_O_4_/AG nanocomposite was successfully synthesized by the self-assembly of Co_3_O_4_ nanoparticles in the AG substrate, followed by heat treatment using resource-rich anthracite as the carbonaceous precursor. The Co_3_O_4_/AG nanocomposite contained uniformly distributed Co_3_O_4_ nanoparticles with a size smaller than 8 nm on the surface of porous graphene, and had a large specific surface area with well-developed mesopores and a high level of nitrogen doping. This unique Co_3_O_4_/AG nanocomposite as an anode for LIBs possessed surface-controlled electrode kinetics and a low internal resistance, delivering a better initial reversible capacity of 845 mAh·g^−1^. Furthermore, a superior cycling stability and rate capability were also achieved. Such an excellent performance results from the unique structure and high conductivity of the nanocomposite. Therefore, the Co_3_O_4_/AG nanocomposite is a potential anode candidate for large-scale application in LIBs.
